# Implementing Integrative Nursing in a Pediatric Setting

**DOI:** 10.3390/children5080103

**Published:** 2018-07-31

**Authors:** Megan E. Voss, Mary Jo Kreitzer

**Affiliations:** Center for Spirituality and Healing, University of Minnesota, Minneapolis, MN 55455, USA; kreit003@umn.edu

**Keywords:** pediatric integrative nursing, program development, pediatric blood and marrow transplant

## Abstract

Pediatric blood and marrow transplantation (BMT) is one of the most challenging allopathic treatments a patient and family can be faced with. A large Midwest academic health center, and leader in pediatric BMT, made the decision in 2013 to incorporate integrative nursing as the care delivery model. Nurses trained in advanced nursing practice and specialized in integrative health and healing performed a deep-dive needs assessment, national benchmarking, a comprehensive review of the literature, and ultimately designed a comprehensive integrative program for pediatric patients and their families undergoing BMT. Four years after implementation, this paper discusses lessons learned, strengths, challenges and next phases of the program, including a research agenda. The authors conclude that it is feasible, acceptable and sustainable to implement a nurse-led integrative program within an academic health center-based pediatric BMT program.

## 1. Introduction

Integrative nursing has emerged as a framework for delivering sound patient care over the past five years. Principles closely aligned with the Institute for Healthcare Improvement’s (IHI) Quadruple Aim can be used to help clinicians work with their patients to achieve better outcomes at lower costs, while at the same time tending to clinician well-being [1]. In the current healthcare environment, clinicians are consistently asked to do more with less. Nurses are caring for patients at much higher acuity levels than ever before, and often with higher nurse-to-patient ratios. Clinician burnout and early exit from the field are reaching alarming levels [2]. Integrative nursing as a care delivery model can be used as a strategy for mitigating the challenges faced by today’s acute care nurses [3,4]. The aspirational vision of the pediatric blood and marrow transplantation (BMT) program’s integrative model of care was to add an additional layer of supportive care that would palliate the patient and family on all dimensions: mind, body and spirit. The vision has remained closely aligned with the IHI Quadruple Aim. The goals of implementing integrative nursing are to improve the care experience of the patient and family, improve symptom management, and enhance the patient and family’s capacity to cope. Additionally, in alignment with the IHI fourth aim, the program aspires to meet the self-care needs of staff from an organizational standpoint and to give providers and staff the tools they need to meet their individual responsibilities for self-care.

## 2. Methods

**Figure 1:**
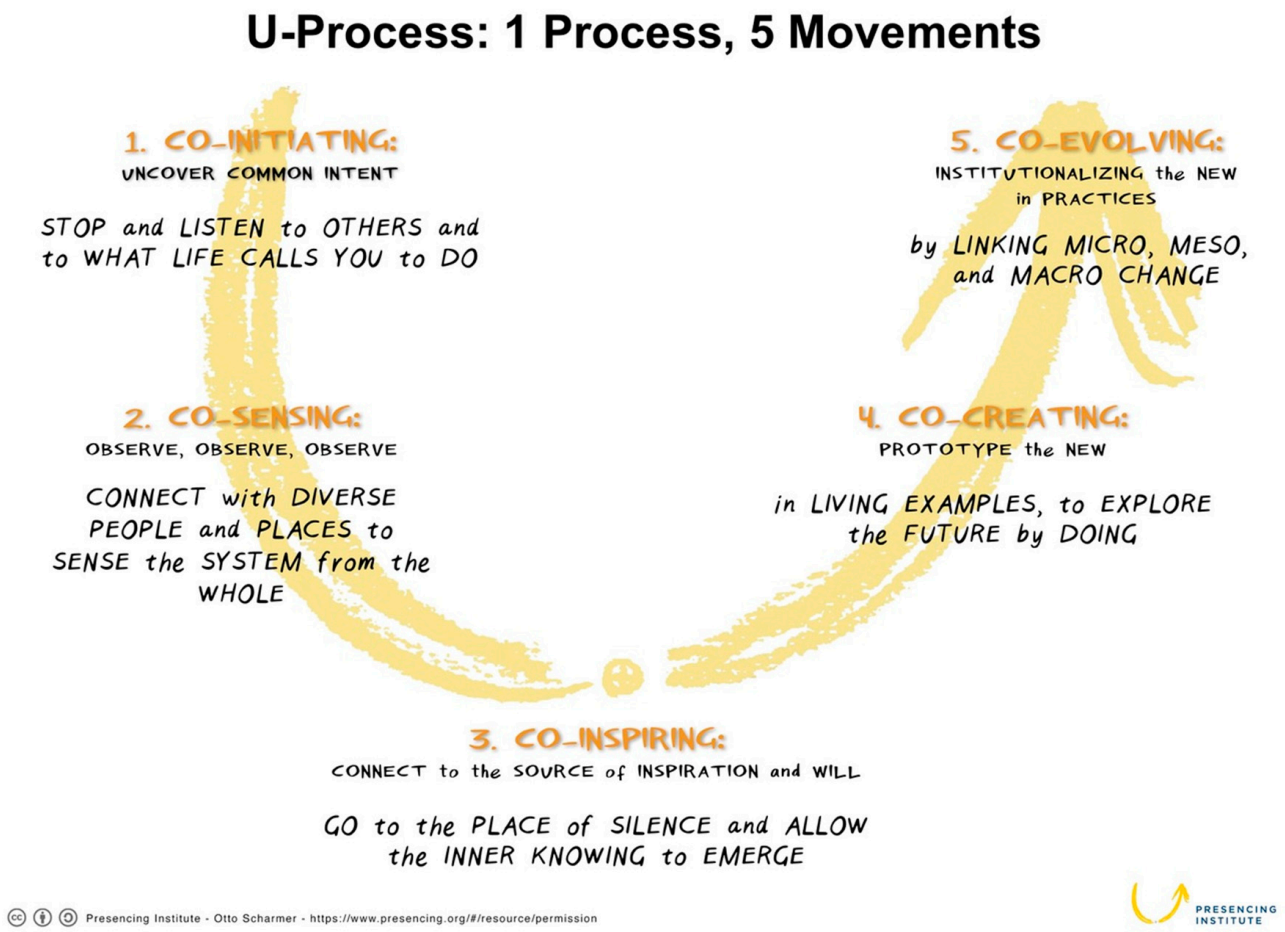
Theory U, reprinted with permission from [5].

A team of advanced nursing practice leaders with specialty training in integrative health and healing performed a four-month long deep-dive needs assessment in order to develop a customized integrative program to complement the pediatric BMT program. Otto Scharmer’s Theory U (Figure 1) served as the framework for the needs assessment. In this model of transformational change, there is a major focus on deeply listening to and understanding the needs of stakeholders in the organization; in this case patients, families, staff and leaders, and to engage them in planning for the desired future [5]. Those five stages are outlined in Figure 1.

### 2.1. Integrative Nursing as a Framework for Practice

The principles of integrative nursing (Table 1) informed the design, implementation and evaluation of the program. Integrative nursing is a whole person, whole system way of caring and healing that shapes and impacts patient care across the continuum. While the first text explicating the principles of integrative nursing was published in 2014, the concepts are timeless, are aligned with major nursing theorists, and date back to the philosophy, values and practices first introduced by Florence Nightingale in the 1800s [3,4].

### 2.2. Program Design and Components

The pediatric BMT integrative therapy program was designed based on the outcomes of a four-month long needs assessment. The needs assessment included the following five components:

- Stakeholder dialogue—Interviews were conducted with clinical and administrative leadership as well as with frontline clinical staff.
- Shadowing practice—Current state of nursing and supportive care staff practice was observed to identify areas of opportunity.
- Prototyping—Experiential and educational sessions were conducted to engage staff and enhance their understanding of integrative modalities.
- Benchmarking—Other pediatric integrative therapy programs were examined through site visits, phone interviews, and extensive internet searches. These were reviewed for best practices.
- Staff survey—Nursing staff (*N* = 32) on the unit were surveyed to assess the knowledge, perceptions and comfort level with integrative modalities and care delivery.

Through this assessment, it was ascertained that the system overall had strong interest in and readiness for an integrative therapy program. The medical director was enthusiastically devoted to supporting this effort both clinically and financially. Many other pediatric hospitals around the nation have established integrative therapy programs; therefore, it was suddenly a market disadvantage to not offer what was becoming a standard of care in the eyes of patients and families [7]. While these strengths culminated to create a culture of momentum, there were still many challenges identified during the needs assessment.

#### 2.2.1. Challenges

- Skepticism from staff—Due to historic start/stop efforts and initiatives that lacked structure, momentum and institutional support, staff doubted the likelihood of ever forming a sustainable, consistent and reliable integrative therapy program. There was distrust of the system and fatigue from working around system failures.
- Perceived barriers—Ranked by nursing staff in descending order: time, lack of skills, lack of knowledge, and comfort with integrative modalities.
- Inconsistencies in infrastructure—Processes, policies, procedure and methods of providing staff education had many variations across the board between the clinic and the hospital settings. These inconsistencies made it challenging to assess what existed, let alone what could be built.
- Lack of clinical expertise—The system did not employ any clinician with the level of training necessary to ensure that safe and effective integrative therapies were being utilized to the fullest potential to meet the needs of patients and families.

Ultimately, it was determined that a historic lack of cohesive institutional effort to form a comprehensive integrative therapy program led to the development of many variations in integrative therapy practices throughout the institution. While this demonstrates a high level of staff dedication to patient needs and adaptability, it also lends itself to inconsistency in standards of practice, policies, procedures and staff education. These types of efforts leave patients and families with minimal access to a consistent, reliable and safe set of services. Despite these challenges, several goals and a common vision emerged as part of the needs assessment process.

#### 2.2.2. Vision

The vision was to create a world-class comprehensive integrative therapy program that would provide a seamless experience for pediatric BMT patients and families from the time of diagnosis through survivorship or bereavement. Additionally, the vision was to align the program with the organizational vision and mission and with national health care goals, including the Quadruple Aim: improving the patient experience, the health of populations, reducing the cost of health care delivery, and improving care team well-being [2]. The program aimed to be viewed as a means to achieving essential health care outcomes by aligning fluidly with the challenges of today’s health care environment. Integrative nursing is a key strategy in improving outcomes, enhancing patient/family satisfaction, and reducing the cost of care delivery, while at the same time improving its quality [3].

#### 2.2.3. Goals

- Improve symptom management.
- Improve patient experience.
- Enhance patient and family resilience and capacity to cope.
- Provide patient and family with tools for incorporating integrative health practices before, during and after hospitalization.
- Improve care team well-being.
- Grow and support integrative health and healing research.
- Embed integrative approaches in every patient’s care plan and every clinician’s workflow.

#### 2.2.4. Recommendations

The recommendations for achieving this vision and these goals were clear, yet extensive and arduous. To build a sustainable program, it would take years to accomplish every essential element. The following list of recommendations was developed five years ago following the initial needs assessment.

- Create an infrastructure and commit resources that are commensurate with the program vision and goals.
  ◦ Hire or appoint an organizational lead.
  ◦ Establish standards of care as well as policies and procedures that support those standards.
  ◦ Create flowsheets and templates in the electronic medical record that not only allow for documentation of integrative approaches to care, but also allow for easy assessment of outcomes.
- Design a robust and comprehensive program evaluation strategy.
- Establish strong staff education and support.
- Offer access to a consistent set of therapeutic approaches that are evidenced-informed and focused on improved symptom management and quality of life.
- Expand patient and family education.
- Develop parent support resources to engage them in self-care and stress management, and to empower them to participate in comforting and caring for their child through the use of integrative approaches.
- Create an integrative health consult service for expertise, leadership and guidance to staff, patients and families.
- Establish an integrative health and healing research program focused on pediatric BMT patients’ needs and clinical outcomes.

The program has three main components: care of patients and their families, staff well-being, and outcome evaluation. The components have been developed and implemented in that order. This order of evolution has been successful for several reasons. Nursing staff often does not recognize the need for self-care, does not identify burnout correctly or timely, or is reluctant to make changes. By implementing care of patients and families first, nurses and other frontline staff experienced secondary benefits of employing integrative strategies in the hospital setting. Nursing frequently received positive feedback from patients and families on the benefits they were experiencing from using integrative modalities.

#### 2.2.5. Care Delivery Model

The framework for the nursing care delivery model comes from the integrative nursing work of Mary Jo Kreitzer and Mary Koithan [3]. The six principles of integrative nursing guide the practice of every nurse in the institution. Additionally, two doctoral prepared nurses with advanced nursing practice study in integrative health and healing lead the delivery of integrative therapy and integrative health consultation. Each new patient receives an integrative health consult with one of the integrative health doctor of nursing practice (DNP) nurses. These consults are triggered in the system to happen automatically for every patient. There is no fee associated with consultation or administration of integrative care. These services are offered to all patients regardless of insurance coverage or ability to pay. During this initial consultation, the patient and family’s historical use of integrative therapies is discussed, an assessment of current or anticipated needs, symptoms and challenges is performed, as is a discussion of current skills, strengths and coping mechanisms. Safety and compatibility of integrative therapies with upcoming treatment regimen is discussed during this consult, and education is provided if a patient is currently using or desiring to use an integrative approach that might pose a safety risk during or immediately following the period of chemotherapy and radiation. Finally, a plan for integrative care during hospitalization is discussed and designed. Upon admission to the BMT unit, patients can choose to receive integrative services daily, as needed, or on a consultation basis only. The most common reasons patient engage in integrative therapies are for pain control, nausea, insomnia, anxiety and benefit of forming a therapeutic relationship with the clinician. Supplementary Materials Table S1 describes the integrative therapies that are offered consistently and reliably on the BMT unit.

### 2.3. Program Evaluation

Entering into the fifth year of the program, data has been gathered showing an uptick in integrative therapy utilization since year one. The authors hypothesize this could be due to several factors: (1) the first year of the program, there was only one full-time clinician dedicated to providing integrative services to patients and families. Currently, there are two-and-a-half full-time equivalent employees providing integrative services. (2) Patients and families affected by childhood cancer, and other rare diseases for which BMT is utilized as a treatment, are very well connected to one another. Patients and families now report having heard about the benefits of integrative therapies from people in their disease-specific communities before ever arriving to the facility. (3) Clinicians have indubitably become more skilled at applying integrative therapies to unique patient populations. Clinicians are skilled at assessing the root cause of a patient’s symptom and applying the therapy that will modify the root cause rather than simply eliminating the symptom. For example, young children often experience nausea and/or vomiting before or after medication administration or in anticipation of chemotherapy. It is important to assess the characteristics of and the circumstances surrounding the nausea and/or vomiting. Often, it is anxiety or anticipatory anxiety that leads to sudden onset nausea and/or vomiting [8]. Mind/body techniques are more effective at mitigating the risk of emesis related to anxiety or a conditioned response to treatment than a pharmaceutical antiemetic [8]. Finally, (4) it is plausible that providers and staff recommend integrative therapies more often and demonstrate more confidence in integrative therapies now than they did four years ago. Once providers and staff began having first-hand observations and experiences with integrative therapies achieving positive results for their patients, they were more likely to recommend them.

The BMT program treats approximately 80 patients per year. Patients are engaged in treatment for a minimum of three to four months. Some patients remain in the system and seek in- and outpatient care intermittently for up to two years. Year one of the program, 51% of BMT patients engaged in integrative therapies. By year two, that number increased to 70%, and at the conclusion of year three, approximately 94% of patients participated in integrative therapies throughout their BMT experience. Patient and family engagement with the integrative therapy team ranges from a one-time consult to daily sessions of 30–60 min. The average patient is seen two or three times per week for approximately 30 min. Over the course of hundreds of integrative therapy sessions over the past four years, the program reports no adverse events. Practice guidelines and safety recommendations have been developed based on available literature, clinician experience, patient report, and occasionally “near misses”.

### 2.4. Emerging Research Agenda

Establishing a research program was one of the eight original goals. This has been the most challenging goal to accomplish and was, by design, delayed for the first few years. Infrastructure needed to be built before clean data collection could occur. Now that a consistent set of services is offered by experienced clinicians, the program is ripe for an integrative research agenda. Several quality improvement surveys and one qualitative study have been completed and played an important role in shaping the research agenda. The first study published by the program examined themes around the usefulness of music therapy [9]. Table 2 outlines the research initiatives that are underway and projects that will be implemented over the next two years.

## 3. Results and Discussion

The integrative therapy program is entering into its fifth year of existence. It has grown and matured since the original design, but remains true to its initial mission, vision and goals. By all objective measures, the program has been deemed a success. Rates of patient engagement and utilization, increase in grant funding and institutional support, and provider and front-line clinician acceptance all speak to the accomplishments of the program over the past four years. The authors conclude that it is both feasible and acceptable to implement a nurse-led integrative program within an academic health center-based pediatric BMT program.

The success and sustainability of the program may be in large part due to nursing leadership. Nurses are uniquely positioned to assess and treat patients from a whole-person, whole-system framework. Nurses are by nature nurturing, caring and attuned to using their own self as a therapeutic intervention. Furthermore, by having advanced nursing practice consultation available, the model is more financially sustainable than a physician-centric integrative consult service. Patients receive competent assessment and continuity of care with nurses trained in integrative modalities accessible to them on a daily basis.

## 4. Conclusions

The work over the past few years has demonstrated that there is a strong clinical and business case for implementing integrative nursing within an acute-care pediatric setting. Patients and families are voicing a desire for this approach to care and there is increasing evidence that integrative approaches can improve clinical outcomes including symptom management [8]. If embedded into the ongoing delivery of care, the implementation of integrative nursing is both feasible and sustainable. It requires investment in education and leadership and perhaps most importantly a culture change that embraces a whole-person, whole-system approach to patient care.

## Figures and Tables

**Figure 2:**
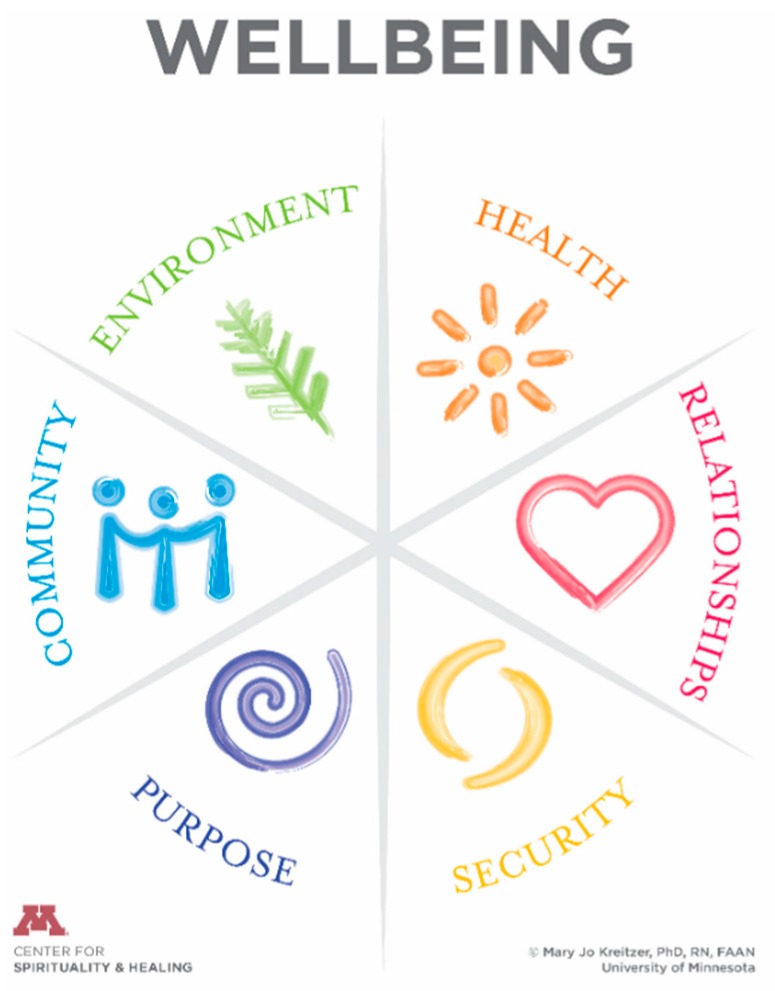
University of Minnesota’s Wellbeing Model [6].

**Table 1:** Principles of integrative nursing [3,6].

Human beings are whole systems inseparable from their environments.
Human beings have the innate capacity for health and well-being.
Nature has healing and restorative properties that contribute to health and well-being.
Integrative nursing is person-centered and relationship-based.
Integrative nursing practice is informed by evidence and uses the full range of therapeutic modalities to support/augment the healing process, moving from least intensive/invasive to more, depending on need and context.
Integrative nursing focuses on the health and well-being of caregivers as well as those they serve.

**Table 2:** Research agenda.

Study Concept	Description
Music therapy as a method to physical rehabilitation	Qualitative data has revealed that even when children are too physically ill or mentally reluctant to participate in physical rehabilitation therapies, many are still motivated to get out of bed and participate in music therapy. It is hypothesized that active participation in music therapy can help achieve physical rehabilitation goals in some patients [9].
Retrospective review of utilization and safety	Data mining will be done to assess the most common therapy used by each age group, adverse reactions in patients with safety considerations such as thrombocytopenia or impaired skin integrity, responses of pain, nausea and anxiety to integrative therapy, and average length and number of visits (inpatient and outpatient) each patient requests.
Self-assessment of change after the implementation of an integrative iherapy ilan of care	The self-assessment of change is a retrospective pre/post assessment that measures a variety of psychosocial indicators. The tool was designed specifically to capture change produced by integrative therapy.
Survivorship well-being for patients and families	Interactive online content based on the University of Minnesota’s Wellbeing Model (Figure 2) will be developed and focused on teenagers and young adults. Virtual support groups will accompany content. Groups will be facilitated by a variety of healthcare professionals with an integrative lens. Data will be collected to assess the impact on overall well-being including indicators such as self-management, perceived stress, quality of life, anxiety, depression and resilience.
Microbiome	This study will examine the effects of chemotherapy and prolonged prophylactic antibiotic use on patients after engraftment and full recovery from pancytopenia. It will also look at the potential for the safe and judicial use of supplements to restore intestinal tissue integrity and the microbiome.
Staff self-care	Continuous data collection efforts have been and will continue to be underway assessing staff burnout and other components of well-being.

## Supplementary Materials

The following are available online at http://www.mdpi.com/2227-9067/5/8/103/s1, Table S1: Integrative therapy availability.
