# Supplementary material for: Implementing Integrative Nursing in a Pediatric Setting
Source: Children (Basel). 2018 Jul 31;5(8):103. doi: 10.3390/children5080103 (PMC6111245; doi:10.3390/children5080103)
Supplement: Supplementary file 1 [file children-05-00103-s001.pdf]

Supplementary Materials

Table S1 Integrative therapy availability.

	Definition	Who Can Administer Therapy?	When is Therapy Available?
**Acupoint**	Umbrella term used to describe both needling & non-needle acupressure and acupuncture techniques.	Non-needling can be performed by nurses who have had education in acupressure. For more extensive consultation, the institution employees a pediatrician who performs acupoint assessment and administers needling acupuncture.	Acupressure can be taught to parents to perform 24/7. Nurses with training may also be available 24/7. Consultation both in-and outpatient is available four days per week.
**Aromatherapy**	The therapeutic use of essential oils both topically and through inhalation to achieve symptom management.	Nurses administer the basic level of products to help with nausea, anxiety, pain and insomnia after completing online training. Advanced consultation is available for wound care and medication interaction concerns.	Products can be obtained via nursing 24/7. Advanced consultation is available Monday-Friday, 8am–6pm.
**Biofeedback**	The use of sensors to measure physiologic response. Through this, the patient learns to recognize and regulate emotional, mental, and physical symptoms.	Integrative health consult nurses.	Integrative health consult nurses available Monday-Friday, 8am–6pm.
**Breathwork**	Teaching school age children, adolescents, teenagers, and young adults, developmentally appropriate ways to manage physical, mental and emotional symptoms through breath regulation.	Nurse employ basic techniques and have access to assistive devices such as pinwheels, bubbles and breathing balls. Child Family Life Specialists do more extensive teaching of skills and ongoing practice with patients and families as do the integrative health consult nurses.	Nurses are available for basic techniques 24/7. Integrative health consult nurses available Monday-Friday, 8am–6pm. Child Family Life Specialists are available 16 h per day.
**Energy therapy**	Reiki and Healing Touch are hands-on, light touch therapies that induce relaxation, enhance sleep, and reduce pain in children.	Nurses can administer energy therapy if they have had training from a recognized source. Integrative health consult nurses are available to provide patients energy therapy when nurses do not have the necessary skill level or time.	Integrative health consult nurses available Monday-Friday, 8am–6pm.

**Massage therapy**	Light touch targeted massage is used to induce relaxation and reduce pain.	Nurses who attended an onsite hands-on training on pediatric massage can administer a variety of techniques. Nursing with less extensive background and training can offer back, foot and hand massage.	Nurses with this skill set may be available 24/7.
		Integrative health consult nurses are available to offer targeted and full body massage when nurses do not have the necessary skill level or time, or when patients are considered high risk (i.e. low platelets or poor skin integrity).	Integrative health consult nurses available Monday-Friday, 8am–6pm.
**Music therapy**	The use of music, interactive or passive, with a credentialed music therapist, to achieve therapeutic goals.	Only a board-certified music therapist can administer music therapy.	Available three days per week for five hours per day to pediatric BMT patients. Most patients receive two 20–45-min sessions per week depending on need, desire, and availability.
**Nutrition/supplement consultation**	Educational and safety-oriented consultation tailored to the pediatric patient undergoing chemotherapy.	Integrative health consult nurse	Integrative health consult nurses available Monday-Friday, 8am–6pm.
**Self-hypnosis training**	Self-directed focus of the mind and imagination to achieve therapeutic goals.	Educational and practice sessions directed by integrative health consult nurse. Sessions are often recorded for patients to use their hypnotic script daily for best results.	Integrative health consult nurses available Monday-Friday, 8am–6pm.
**Yoga**	Gentle yoga provides physical, mental and emotion benefits through meditation and physical engagement.	Integrative health consult nurse.	Integrative health consult nurses available Monday-Friday, 8am–6pm.
